# Diseases of the Gums

**Published:** 1873-04

**Authors:** H. S. Miller


### SELECTED ARTICLES.

---

### ARTICLE IV.

### *Diseases of the Gums.*

BY DR. H. S. MILLER.

The more immediate diseases to which the gums are liable arise from local irritations, a common one of which, and probably the most frequently met with, is a deposit of salivary calculus, the treatment of which consists in its removal. And here let me urge the necessity of its entire removal, and the thorough polishing of the tooth. All know the effect of even a small scale of tartar, especially the dark, hard variety, deposited far up under the gum, sometimes to the very apex of the root, the result of which is constant irritation, terminating in ulitis, and possibly loss of the tooth.

As before stated, thorough removal is necessary, and with good effect; where the gums are badly inflamed, a wash of diluted carbolic acid or some astringent may be applied.

The gums are indirectly liable to some disease, a proper diagnosis of which is very uncertain. These diseases, of course, require systematic treatment, and it were well for us, as a profession, if we understood better than we do the whole physical organism, a part of which we are constantly operating upon.

For example, the difficulties arising from a too free use of the different mercurial preparations are very similar in their nature to scorbutus. The same local remedies would perhaps apply in either case; but in systematic treatment quite different remedies are necessary. For ulitis, the result of mercurial impressions, tincture of iodine, applied locally, is very prompt in its action. Indeed, this remedy is almost indispensable in most of the diseases of the gums.

Chlorate of potassa and sub-borate of soda, though not always quite so active, are also beneficial. Many of the diseases to which the gums are subject have their origin in tertiary syphilis, the periosteum of the alveoli being, either directly or indirectly, involved. As an example, though, perhaps, not entirely pertinent to the subject, I have a patient now under treatment, sent me by a medical practitioner, who had had no apparent trace of this disease for twelve years, when suddenly it made its appearance in the gums surrounding the left cuspid, lateral and central incisors. I found the immediate cause of the diseased condition of the gums to arise from necrosis of the alvoeli of these teeth. My treatment was first to excise this necrosed bone, and after daily syringing the parts involved, to apply dilute tincture of iodine. Result not satisfactory. Then tried carbolic acid with, however, no better effect. Finally resorted to strong solution of nitrate of silver, and with such success that granulation has taken place, and the case nearly cured. Meanwhile iodide of potassium was given internally.

A very frequent cause of disease of the mucous membrane of the mouth and gums is the wearing of artificial dentures improperly adjusted, or of improper material. Especially is this the case where the teeth are mounted on the Goodyear-Cummings-Bacon patent vulcanized rubber. The only remedy is to remove the plate, apply astringents, and curse Bacon and the day rubber was first introduced to the profession.

As regards absorption of the alveoli, recession of the gums and deposition of salivary calculus, no reliable treatment has as yet been put forth of a permanent character. In this connection there is an almost unlimited field for the dental practitioner. Unfortunately with far too many, pecuniary success is the only consideration. As Holland says with regard to our country, so say I with regard to our profession: "God give us men! A time like this demands strong minds, great hearts, true faith and ready hands."—*Dental Times.*

